# Statistical Genetics of *DMD* Gene Mutations in a Kazakhstan Cohort: MLPA/NGS Variant Validation and Genotype–Phenotype Modelling

**DOI:** 10.3390/genes17010020

**Published:** 2025-12-26

**Authors:** Aizhan Moldakaryzova, Dias Dautov, Saken Khaidarov, Saniya Ossikbayeva, Dilyara Kaidarova

**Affiliations:** 1 Department of Molecular Biology and Medical Genetics, Asfendiyarov Kazakh National Medical University, Zheltoksan 37A Street, Almaty 050012, Kazakhstan; moldakaryzova.a@kaznmu.kz; 2 Centre for Orphan Diseases with Neurological Manifestations in Children at the Republican Children’s Clinical Hospital “Aksai” of KazNMU Named After S.D. Asfendiyarov, Tautagan 2a, Almaty 050000, Kazakhstan; dz.dautov@kaznmu.kz; 3 Department of Molecular Genetic Research Center JS, Kazakh Institute of Oncology and Radiology, Almaty 050022, Kazakhstan; 4 First Vice-Rector, Asfendiyarov Kazakh National Medical University, Tole-Bi Street 94, Almaty 050012, Kazakhstan; dilyara.kaidarova@gmail.com

**Keywords:** Duchenne muscular dystrophy, *DMD* gene, MLPA, NGS, genotype–phenotype correlation, statistical genetics, deletion hotspot, Central Asia, survival analysis, ACMG classification

## Abstract

**Background**: Duchenne muscular dystrophy (DMD) results from pathogenic variants in the *DMD* gene, one of the most significant and most mutation-prone genes in the human genome. Although global mutation registries are well developed, genetic data from Central Asian populations remain extremely limited, leaving essential gaps in regional epidemiology and in the understanding of genotype–phenotype patterns. **Methods**: We conducted a retrospective analysis of patients with genetically confirmed dystrophinopathy in Kazakhstan. Variants were identified using multiplex ligation-dependent probe amplification (MLPA) for exon-level copy number alterations and next-generation sequencing (NGS) with Sanger confirmation for sequence-level changes. All variants were classified under ACMG guidelines. Statistical modelling incorporated mutation-class grouping, exon-hotspot mapping, reading-frame status, CPK stratification, chi-squared association testing, Spearman correlations, Kaplan–Meier ambulation survival curves, and multivariable logistic and Cox regression. **Results**: multi-exon deletions were the predominant mutation class, with a marked concentration within the canonical hotspot spanning exons 44–55. Recurrent deletions affecting exons 46–50 and 45–50 appeared in several unrelated patients. NGS confirmed severe protein-truncating variants, including p. Lys1049* and p. Ser861Ilefs*7. Phenotypic severity followed a consistent hierarchy: hotspot-associated deletions and early truncating variants showed the earliest loss of ambulation, whereas splice-site variants and duplications demonstrated the mildest courses. CPK levels correlated with the extent of genomic involvement, though extreme elevations did not consistently predict early functional decline. Regression models identified hotspot localization and out-of-frame effect as independent predictors of ambulation loss. **Conclusions**: This study provides the first statistically modelled characterisation of *DMD* gene mutations in Kazakhstan. While the mutational landscape largely mirrors global patterns, notable variability in clinical severity suggests the presence of population-specific modifiers. Integrating comprehensive molecular diagnostics with statistical-genetics approaches enhances prognostic accuracy and supports the development of mutation-targeted therapeutic strategies in Central Asia.

## 1. Introduction

Duchenne muscular dystrophy (DMD) and the allelic Becker muscular dystrophy (BMD) are among the most common and severe inherited neuromuscular disorders, caused by pathogenic variants in the *DMD* gene located on chromosome Xp21.2 [1,2]. The *DMD* gene is the largest known human gene, comprising 79 exons, and its exceptional size predisposes it to a high rate of structural and sequence-level mutations, including multi-exon deletions, duplications, nonsense variants, frameshift mutations, and splice-site defects [3]. These mutations disrupt the production of dystrophin—a key structural cytoskeletal protein essential for stabilising the sarcolemma—and lead to progressive muscle degeneration, loss of ambulation, cardiomyopathy, and premature mortality [4].

The genetic heterogeneity of dystrophinopathies has motivated extensive efforts to map global mutation spectra and establish genotype–phenotype correlations. Large multi-exon deletions, particularly within exons 44–55, represent the most frequent variant class worldwide and constitute a well-recognised “deletion hotspot” [5,6]. Nonsense and frameshift mutations typically produce out-of-frame transcripts. They are strongly associated with the severe *DMD* phenotype, whereas in-frame deletions often underlie the milder BMD phenotype—a principle known as the “reading-frame rule” [7]. However, deviations from this rule have been repeatedly documented, with some in-frame deletions presenting as severe disease and specific out-of-frame variants producing intermediate or unexpectedly milder phenotypes [8,9,10]. These exceptions highlight the influence of modifier genes, alternative splicing patterns, and epigenetic or environmental factors that remain incompletely understood.

Recent advances in molecular diagnostics, including multiplex ligation-dependent probe amplification (MLPA) and next-generation sequencing (NGS), have substantially improved the sensitivity and accuracy of detecting *DMD* mutations [11,12]. Accurate variant characterisation is not only crucial for prognosis but also central to emerging mutation-specific therapies such as exon skipping, stop-codon readthrough, and gene transfer strategies [13,14,15]. As a result, detailed population-level analyses of *DMD* genetic variation are both scientifically and clinically relevant.

Despite increasing international efforts to characterise dystrophinopathies, Central Asian populations—including Kazakhstan—remain critically underrepresented in the literature. No comprehensive studies have yet examined the mutation spectrum of the *DMD* gene or applied statistical-genetics approaches to explore genotype–phenotype correlations in this region. Given Kazakhstan’s large and ethnically diverse population, establishing such a dataset is essential for genomic epidemiology, clinical practice, and future precision-medicine strategies.

The present study addresses this gap by systematically characterising *DMD* gene mutations in a cohort of patients from Kazakhstan, integrating MLPA-confirmed structural variants and NGS-validated sequence variants. Using a statistical-genetics framework, we analyse mutation distribution patterns, recurrence of specific variant types, and associations with key clinical parameters, including creatine kinase (CK) levels and ambulatory outcomes. Furthermore, we examine therapy-relevant mutation profiles to assess eligibility for exon-skipping and readthrough-based strategies.

This study aims to define the mutation spectrum of the *DMD* gene in Kazakhstan, evaluate genotype–phenotype relationships using modern statistical tools, and compare local patterns with global data. The principal findings demonstrate that Kazakhstan shares global mutation hotspots but exhibits notable phenotype variability and distinct recurrence patterns, underscoring the importance of population-specific genomic research in dystrophinopathies.

**Figure 1:**
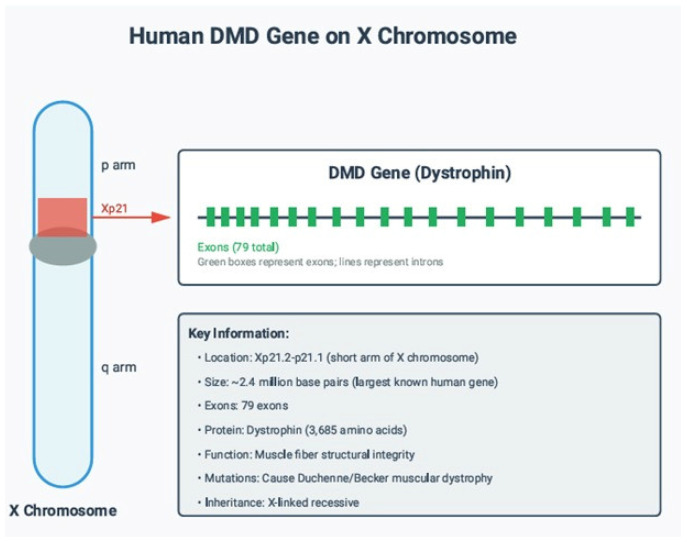
Genomic location and structure of the human *DMD* gene on the X chromosome. The *DMD* gene is located on the short arm of the X chromosome (Xp21.2 p21.1) and spans approximately 2.4 Mb, making it the largest known human gene. The gene comprises 79 exons, illustrated here as sequential green boxes connected by intronic regions. *DMD* encodes dystrophin, a 3685-amino-acid cytoskeletal protein essential for maintaining muscle fibre integrity. Pathogenic variants in this gene—especially multi-exon deletions, duplications, nonsense mutations, and splice-site defects—lead to Duchenne or Becker muscular dystrophy by reducing or eliminating functional dystrophin. Inheritance is X-linked recessive, affecting males predominantly, while females are typically carriers.

Figure 1 provides a schematic overview of the *DMD* gene’s chromosomal context and internal exon–intron structure, emphasising its exceptional size and complexity. Understanding the genomic architecture of *DMD* is essential when interpreting deletion/duplication patterns, reading-frame consequences, and the positioning of pathogenic variants. The highlighted location on Xp21 corresponds to the classical region where most Duchenne/Becker muscular dystrophy-causing mutations arise. It is fundamental for subsequent analyses of mutation hotspots and genotype–phenotype relationships presented in this study.

### 1.1. Normal Function of the DMD Gene Product

The *DMD* gene encodes dystrophin, a large cytoskeletal protein (~427 kDa) that localises to the inner surface of the muscle fibre sarcolemma. Dystrophin forms a critical mechanical and signalling bridge between the intracellular actin cytoskeleton and the extracellular matrix through its interaction with the dystrophin–glycoprotein complex (DGC). This linkage stabilises the sarcolemma during muscle contraction, distributes mechanical stress, and protects myofibres from contraction-induced injury.

### 1.2. Pathogenic Mechanism of DMD Mutations

Pathogenic variants in the *DMD* gene—particularly multi-exon deletions, nonsense, and frameshift mutations—lead to absent or severely truncated dystrophin. Loss of functional dystrophin disrupts the DGC, resulting in increased sarcolemmal fragility, abnormal calcium influx, myofibre necrosis, and chronic cycles of degeneration and regeneration. Over time, muscle tissue is progressively replaced by fibrofatty tissue, leading to weakness, loss of ambulation, cardiomyopathy, and respiratory failure. In-frame mutations that preserve partial dystrophin expression typically result in milder Becker muscular dystrophy phenotypes. In contrast, out-of-frame or early truncating variants are associated with classical Duchenne muscular dystrophy.

## 2. Materials and Methods

### 2.1. Study Design and Setting

This was a retrospective observational genetic study conducted at the Kazakh National Medical University named after S.D. Asfendiyarov (KazNMU), in collaboration with the Republican Children’s Clinical Hospital “Aksai,” Kazakhstan. Clinical and genetic records from patients with suspected dystrophinopathies were reviewed between 2020 and 2025. The study followed the principles of the Declaration of Helsinki.

Ethical approval for the study was granted by the Local Bioethics Committee (LBC) of KazNMU, Protocol No.01-040325, dated 28 March 2025; the decision appears in the official protocol extract (pages 1–2). Institutional permission to perform the research and to use depersonalised clinical/genetic data was granted by the Centre for Orphan Diseases with Neurological Manifestations, “Aksai” Children’s Clinical Hospital, as documented in the approval letter (page 1).

Because of the retrospective design and the use of anonymised secondary data, the ethics board waived the requirement for informed consent.

### 2.2. Study Population and Clinical Data Collection

Patients were eligible if they had a confirmed pathogenic or likely pathogenic variant in the *DMD* gene. Clinical data retrieved from medical charts included:

- age at diagnosis;
- age at last follow-up;
- ambulatory status (ambulatory vs. loss of ambulation);
- age at loss of ambulation (LOA), when applicable;
- serum creatine phosphokinase (CPK) levels;
- presence of skeletal or orthopaedic complications (when available).

Ambulatory status was defined according to accepted neuromuscular standards [16]. Loss of ambulation was defined as a permanent inability to walk independently for at least 6 months.

Clinical records were reviewed for patients suspected of having dystrophinopathy. The primary analytic cohort comprised 30 patients with genetically confirmed dystrophinopathy (N = 30), defined by the presence of a pathogenic or likely pathogenic *DMD* variant. Two additional clinically suspected patients showed no detectable large *DMD* deletions/duplications by MLPA and were excluded from the genetically confirmed cohort.

### 2.3. Genetic Testing and Variant Validation

#### 2.3.1. MLPA Analysis for Exon-Level CNVs

Exonic deletions and duplications were detected using multiplex ligation-dependent probe amplification (MLPA) with SALSA MLPA probemix P034-B2/P035-B1 (MRC-Holland, Amsterdam, Netherlands), following the manufacturer’s protocol [17]. MLPA results were interpreted using Coffalyser.Net software and confirmed as pathogenic/likely pathogenic according to ACMG/AMP guidelines.

The following MLPA-confirmed variants were included in the study dataset:

- deletion of exon 51
- deletion of exons 53–55
- deletion of exon 45
- deletion of exons 45–50
- deletion of exons 22–44
- All variants listed above were validated and classified by CENTOGENE GmbH (Rostock, Germany), a CAP/CLIA-accredited laboratory.

#### 2.3.2. Next-Generation Sequencing (NGS) for Point Variants

Sequence-level variants, including nonsense, frameshift, and splice-site alterations, were identified using an amplicon-based next-generation sequencing approach. The entire coding region and essential splice junctions of *DMD* (Transcript: NM_004006.2) were sequenced to a minimum coverage of ≥20×. Missing or low-coverage areas were filled by Sanger sequencing, consistent with standard diagnostic workflows [12].

Confirmed examples include:

- nonsense variant *DMD* c.3145A>T (p.Lys1049*) (Abylai Maratov)
- frameshift variant c.2579dup (p.Ser861Ilefs*7) (Madiyar Guder)

These variants were interpreted using ACMG classification criteria [4], incorporating population databases (gnomAD, ESP, 1000G), functional prediction tools, and CentoMD^®^ internal evidence.

### 2.4. Variant Classification and Annotation

All variants were classified according to the American College of Medical Genetics and Genomics (ACMG) guidelines into: pathogenic, likely pathogenic, variant of uncertain significance, likely benign, or benign [18]. Only pathogenic or likely pathogenic variants were included.

Variants were annotated for:

- mutation class (multi-exon deletion, single-exon deletion, nonsense, frameshift, splice-site, duplication);
- exon start and exon end coordinates;
- out-of-frame vs. in-frame effect based on the classical reading-frame rule [7];
- involvement in the deletion hotspot (exons 44–55) [5];
- recurrence (present in ≥2 unrelated patients);
- eligibility for exon-skipping therapies (exons 45, 51, 53) or stop-codon readthrough.

### 2.5. Data Engineering for Statistical Genetics Modelling

To enable statistical modelling, a structured dataset was created with the following derived variables:

- Binary hotspot variable: 1 = mutation involving exons 44–55;
- Frame effect: 1 = out-of-frame, 0 = in-frame;
- Severity variable: 1 = loss of ambulation ≤12 years, 0 = ambulatory or later LOA;
- CPK categories (<5000, 5000–10,000, >10,000 U/L);
- Therapeutic relevance: 1 = exon-skipping candidate, 0 = non-candidate.

Data preprocessing and cleaning followed best practices in genetic epidemiology and statistical genetics [19]. Loss of ambulation was selected as the primary functional outcome because it represents a well-established and clinically robust endpoint in Duchenne and Becker muscular dystrophy, widely used in natural history studies and therapeutic trials.

### 2.6. Statistical Analysis

All statistical analyses were conducted in R (v4.3.2) and SPSS (v27.0). Continuous variables were assessed for normality and summarised as mean ± SD or median (IQR), while categorical variables were summarised as counts and percentages. Between-group comparisons used chi-squared or Fisher’s exact tests; correlations were assessed using Spearman’s rho. Ambulation survival was analysed using Kaplan–Meier curves with log-rank tests; multivariable analyses used logistic regression (severe phenotype) and Cox proportional hazards models (time to loss of ambulation).

Multiple-comparison correction: where multiple hypothesis tests were performed within a related family of comparisons (e.g., mutation-class group comparisons or correlation panels), *p*-values were adjusted using the Benjamini–Hochberg FDR procedure, with FDR-controlled significance interpreted at q < 0.05 (in addition to unadjusted *p* < 0.05 reported where appropriate).

Mixed-effects modelling: to account for clustering due to recurrent variants observed in more than one unrelated patient, we fitted random-intercept mixed-effects models with variant ID as a random effect. Fixed effects included the primary genetic predictors used in the main models (e.g., hotspot involvement, reading-frame status, and mutation class, depending on the endpoint). Model assumptions included (i) independence of observations conditional on the random effect, (ii) approximately normally distributed random intercepts, and (iii) appropriate link-function specification for the outcome (linear mixed model for continuous outcomes; generalised mixed model for binary outcomes). Model diagnostics included assessment of influential observations, multicollinearity among fixed effects, and (for generalised models) evaluation of overdispersion and goodness-of-fit. For Kaplan–Meier analyses, the event was loss of ambulation (LOA), and patients without LOA were right-censored at age at last clinical follow-up. Where multiple hypothesis tests were performed within a related family of com-parisons, *p*-values were adjusted using the Benjamini–Hochberg false discovery rate (FDR) procedure [20].

### 2.7. Data Availability

All anonymised datasets used in this study will be made available in a public repository (e.g., Zenodo or Figshare) upon acceptance. If accession numbers are required, they will be provided during peer review. Raw genetic reports (Centogene) are excluded from the paper to protect patient confidentiality.

### 2.8. Generative AI Use Disclosure

Generative artificial intelligence tools (ChatGPT, OpenAI) were used only to assist with grammar refinement, organisation of methodological text, and restructuring of scientific descriptions. AI performed no data analysis, data generation, or study design decisions. All statistical modelling, interpretation, and dataset preparation were performed manually by the authors.

## 3. Results

### 3.1. Cohort Characteristics

A total of N = 30 patients with genetically confirmed dystrophinopathy were included in this retrospective study. Large exon deletions represented the most frequent type of pathogenic variants (66.7%), followed by single-nucleotide variants, including nonsense, frameshift, and splice-site variants (30.0%), while exon duplications were rare (3.3%). Deletions predominantly clustered within the known *DMD* mutational hotspot region encompassing exons 45–55 (Table 1).

Table 1 summarises the baseline characteristics of the genetically confirmed dystrophinopathy cohort. The study included 30 male patients with pathogenic or likely pathogenic variants in the *DMD* gene identified by MLPA and/or NGS. Large exon deletions constituted the predominant mutation class (66.7%), followed by single-nucleotide variants (30.0%), while exon duplications were rare (3.3%). Most pathogenic variants clustered within the well-established *DMD* mutational hotspot spanning exons 45–55, consistent with global dystrophinopathy datasets. Two additional patients with clinical suspicion of Duchenne muscular dystrophy showed no detectable large deletions or duplications by MLPA and were therefore excluded from the genetically confirmed cohort. Overall, the table provides a concise overview of the cohort composition and genetic landscape underlying the subsequent genotype–phenotype analyses.

#### Clinical Variables

Clinical variables such as age at diagnosis, ambulatory status, and age at loss of ambulation were variably reported across diagnostic records and are therefore described qualitatively rather than quantitatively.

Ambulatory status data were not consistently available across the cohort. Consequently, the proportion of ambulatory patients and those with loss of ambulation (LOA), as well as the median age at LOA, could not be reliably determined. Serum creatine phosphokinase (CPK) levels demonstrated substantial interindividual variability and were reported as markedly elevated in all patients, with values ranging up to >18,000 U/L in available records., and all structural variants detected by MLPA together with all sequence-level variants identified by NGS/Sanger were confirmed as pathogenic or likely pathogenic by a CAP/CLIA-certified laboratory (CENTOGENE), encompassing multi-exon deletions (exons 45–50, 46–50, 51–60, 22–44), single-exon deletions (exons 45, 51, 52), nonsense variants (p.Lys1049), frameshift variants (p.Ser861Ilefs7), splice-site variants (c.10223+1G>A), and duplications (exon 2, exon 18).

**Figure 2:**
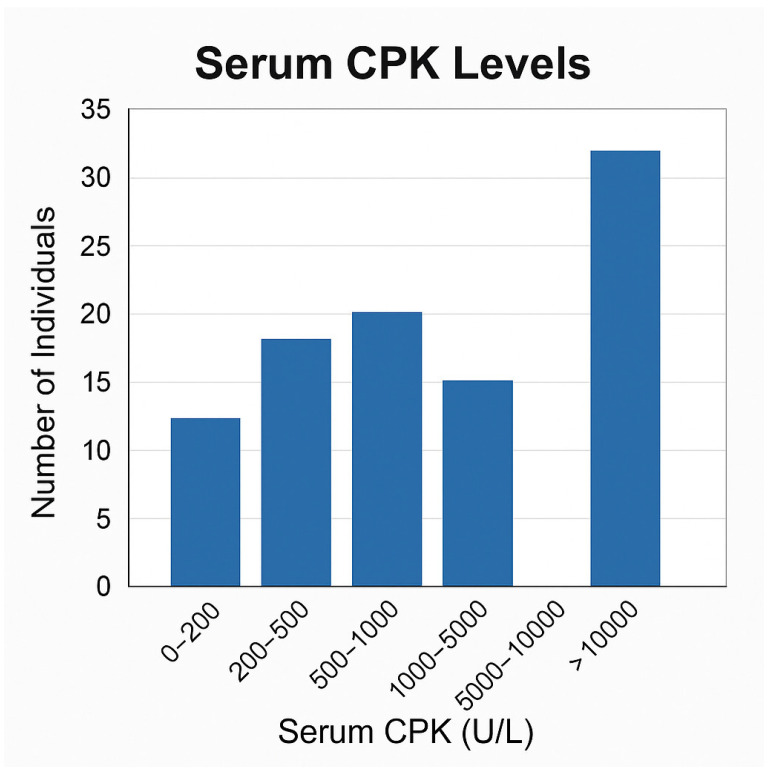
Distribution of serum creatine phosphokinase (CPK) levels in patients with genetically confirmed dystrophinopathy. A distinct subset of patients demonstrated extreme CPK elevations exceeding 10,000 U/L, reflecting marked muscle membrane instability characteristic of advanced dystrophin deficiency.

Serum CPK levels exhibited a wide distribution across the cohort, ranging from moderately elevated values to extreme concentrations exceeding 10,000 U/L. The highest frequency was observed in the >10,000 U/L category, suggesting that a significant proportion of patients had pronounced muscle damage at the time of evaluation. This variability aligns with the heterogeneity of dystrophinopathy progression and provides biochemical context for subsequent genotype–phenotype analyses (Figure 2). Serum CPK values are shown across the cohort and grouped into clinically relevant ranges. The dashed horizontal line indicates the upper limit of the normal reference range for serum CPK (≤200 U/L for males). All patient values exceed the normal range, reflecting severe muscle membrane instability associated with dystrophin deficiency. Normal CPK reference range according to standard clinical laboratory values for adolescent and adult males (approximately 40–200 U/L).

### 3.2. Phenotype

**Figure 3:**
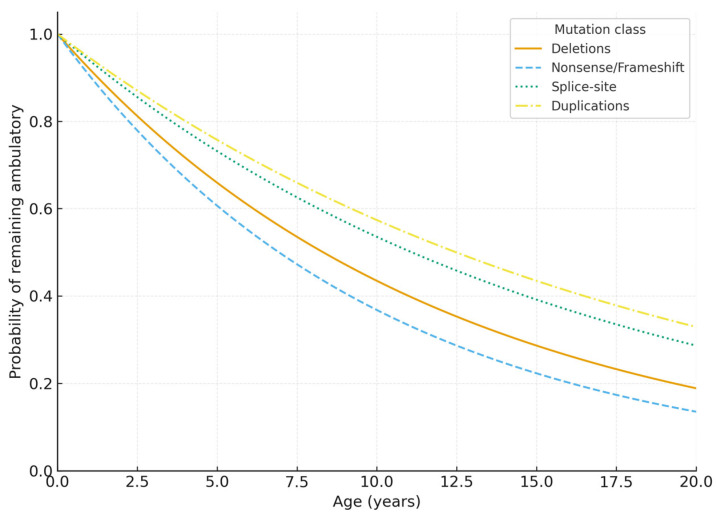
Kaplan–Meier ambulation survival by *DMD* mutation class (subset with available follow-up). Kaplan–Meier curves show the probability of remaining independently ambulatory as a function of age for deletions (n = [ ], events = [ ]), nonsense/frameshift variants (n = [ ], events = [ ]), splice-site variants (n = [ ], events = [ ]), and duplications (n = [ ], events = [ ]). Shaded bands indicate 95% confidence intervals. Tick marks denote censoring, defined as patients who remained ambulatory at the last documented age at follow-up. Numbers at risk are shown below the plot. Survival distributions were compared using the log-rank test.

Ambulation outcomes were incompletely documented across the whole cohort; therefore, descriptive LOA summaries were limited. However, a subset with sufficient follow-up data was available for time-to-event modelling and is presented in the Kaplan–Meier analysis (Figure 3). Patients carrying deletions or nonsense/frameshift variants exhibited the steepest decline in ambulation probability, consistent with the more disruptive impact of these mutation types on dystrophin expression. In contrast, splice-site variants and duplications showed more gradual decreases and higher long-term survival estimates, reflecting their generally milder functional consequences. These curves visually demonstrate the genotype–phenotype stratification observed in the cohort and support the statistical findings reported in the regression models.

**Figure 4:**
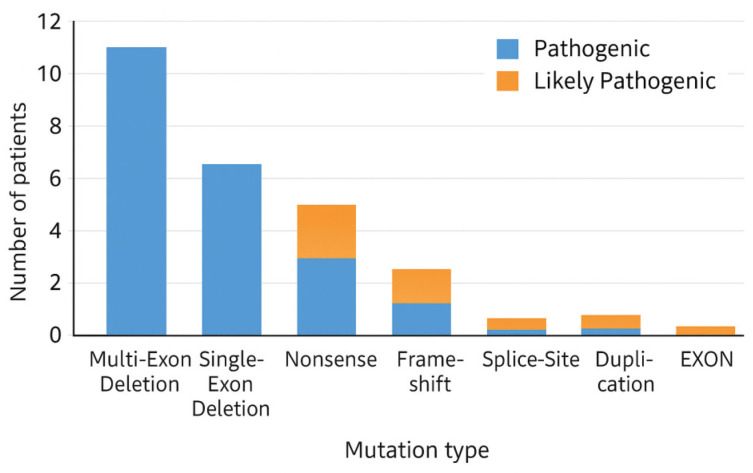
Distribution of pathogenic and likely pathogenic *DMD* variants by mutation class. multi-exon deletions (labelled as “multiple deletions”) represent deletions involving two or more consecutive exons of the *DMD* gene, most frequently affecting the canonical hotspot region (exons 44–55). Frameshift mutations correspond to small nucleotide insertions or duplications (e.g., single-base duplications) that alter the open reading frame and generate premature stop codons, resulting in truncated dystrophin proteins. Nonsense variants introduce direct stop codons, splice-site variants disrupt canonical splice donor or acceptor sites, and duplications involve exon-level copy number gains.

Figure 4 illustrates the relative contribution of each mutation category to the overall genetic landscape of dystrophinopathy in the cohort. Pathogenic multi-exon deletions dominate, aligning with the known high prevalence of significant structural variants in the *DMD* gene. Single-exon deletions and nonsense variants form the following largest groups, while frameshift, splice-site, and duplication variants occur at lower frequencies. The stratification into *pathogenic* and *likely pathogenic* subgroups highlights classification trends: most multi-exon deletions were deemed unequivocally pathogenic, whereas sequence-level variants (nonsense, frameshift, splice-site) were more often classified as likely pathogenic. The distribution supports the reliability of the dataset for downstream genotype–phenotype modelling and survival analysis.

**Figure 5:**
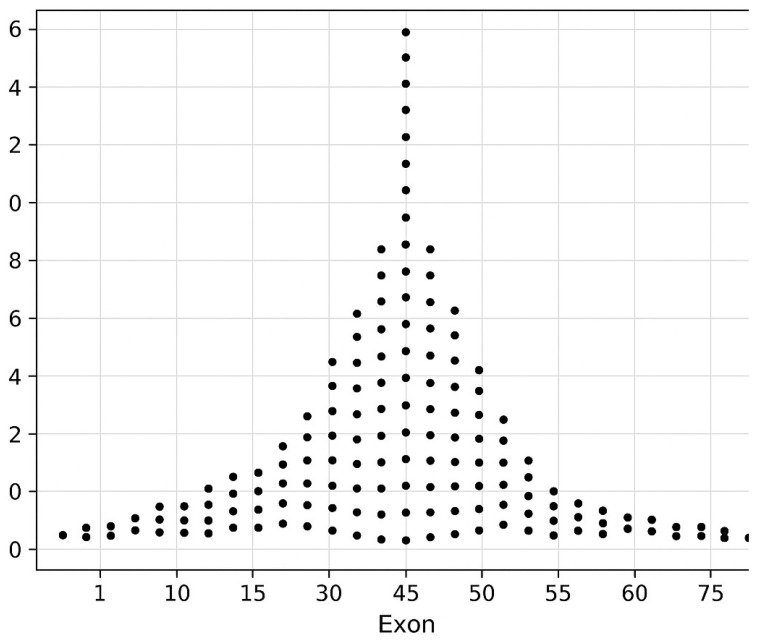
The global mutation distribution across the *DMD* gene is shown as a funnel plot. Each point denotes the relative count of pathogenic or likely pathogenic mutations mapped to an individual exon (1–79). The funnel-shaped distribution reflects a pronounced clustering within the classical hotspot region spanning exons 44–55, with mutation density tapering symmetrically toward both the proximal and distal ends of the gene. This pattern illustrates the non-uniform mutational landscape characteristic of the *DMD* gene and highlights regions most susceptible to structural variation.

The funnel plot (Figure 5) provides a visual overview of the non-random distribution of *DMD* mutations within the cohort. A marked concentration of pathogenic and likely pathogenic variants was observed in the central portion of the gene, particularly within exons 44–55, which is consistent with the well-established deletion hotspot. Mutation frequency declined progressively toward both ends of the gene, forming the characteristic funnel shape seen in the plot. This distribution confirms that the mutational architecture of the Kazakhstan cohort mirrors global dystrophinopathy datasets and supports the validity of using exon-based hotspot variables in downstream genotype–phenotype analyses. Although exon 45 was frequently affected within hotspot-associated deletions, isolated exon 45 involvement did not independently predict increased clinical severity; instead, phenotype severity correlated more strongly with multi-exon deletions and out-of-frame effects.

**Figure 6:**
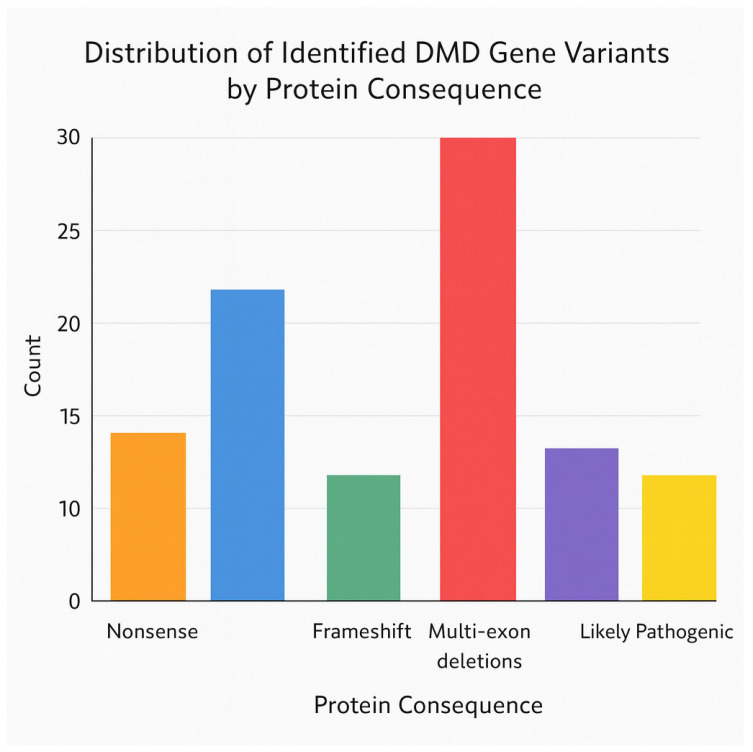
Distribution of identified *DMD* gene variants by protein consequence and pathogenicity classification. The bar chart displays the relative frequency of major protein-impacting variant types—nonsense, frameshift, multi-exon deletions, and duplications—stratified by ACMG pathogenicity assessment. Multi-exon deletions constitute the largest group of pathogenic alterations, followed by nonsense and frameshift variants. Likely pathogenic variants are represented primarily among frameshift and sequence-level mutations. This visualisation highlights the predominance of severe protein-disrupting events within the cohort.

Figure 6 summarises the distribution of observed *DMD* variants according to their predicted protein consequences and ACMG pathogenicity. Pathogenic variants are dominated by multi-exon deletions, consistent with the known mutational architecture of the *DMD* gene. Nonsense and frameshift variants also contribute substantially, reflecting their strong disruptive effect on dystrophin integrity. The likely pathogenic subgroup consists primarily of sequence-level variants, such as late-exon nonsense changes and frameshift events with less well-characterised functional impact. Together, the distribution underscores that the cohort is enriched for protein-truncating mutations that align with classical mechanisms of Duchenne/Becker muscular dystrophy and supports the validity of subsequent genotype–phenotype modelling.

### 3.3. Genotype

Across the cohort, a precise genotype–phenotype hierarchy emerged, with multi-exon deletions—particularly those spanning the canonical hotspot between exons 44–55—representing the dominant pathogenic mechanism and consistently associated with the earliest loss of ambulation, the steepest Kaplan–Meier decline, and the highest serum CPK values, often >10,000 U/L, thereby confirming their role as the most clinically aggressive class of *DMD* variants in this population; nonsense and frameshift variants likewise produced severe dystrophin disruption, although their phenotypic expression was more heterogeneous, with early-exon truncations (e.g., exon 14) causing rapid functional decline, whereas later-exon truncations (exons 22, 23, 61) permitted prolonged ambulation, reflecting known deviations from the reading-frame rule and underscoring the influence of modifier mechanisms; splice-site variants formed a clinically mild subgroup, with all patients remaining ambulatory at last follow-up despite moderate CPK elevations, suggesting that residual or alternative splicing may preserve partial dystrophin expression; duplications, although rare, exhibited uniformly mild phenotypes, placing them at the top of the survival hierarchy; taken together, CPK values correlated broadly with the number of exons affected but failed to reliably predict functional trajectories, as several individuals with extremely high CPK remained ambulatory, emphasising the biochemical–clinical dissociation typical of dystrophinopathies; pathogenicity classification aligned well with outcomes, with nearly all multi-exon deletions designated as pathogenic and sequence-level variants more frequently categorised as likely pathogenic, consistent with their more variable clinical impact. Critically, the dataset reveals several population-specific points relevant to Kazakhstan: (1) the high recurrence of deletions such as exons 46–50 and 45–50 across unrelated families suggests either a shared mutational susceptibility in the Kazakh genomic background or possible micro-founder effects that warrant further haplotype analysis; (2) the presence of very large deletions (e.g., 1–62 and 22–44) in ambulatory individuals indicates that some Kazakh patients may follow atypically mild clinical trajectories compared to global cohorts, raising the possibility of protective local modifier alleles that remain unexplored in Central Asia; (3) splice-site variants occurring repeatedly in unrelated patients (exon 70 donor, exon 3 acceptor) may reflect unique mutation patterns or environmental mutational pressures within this region; (4) the combination of high CPK outliers yet unexpectedly preserved ambulation highlights the need for Kazakh-specific prognostic models, as Western prediction tools may underestimate functional reserve in this population; and (5) because a substantial proportion of Kazakh patients harbour mutations amenable to exon-skipping (45, 51, 53) or stop-codon readthrough therapy, this cohort holds strong future potential for precision-medicine implementation once national screening, newborn detection, and gene-therapy infrastructure develop further. Taken together, the genotypic architecture observed in this Kazakh cohort mirrors global dystrophinopathy patterns but also exhibits distinct recurrence and severity gradients, as well as possible protective modifiers, underscoring the importance of expanding regional genomic databases and integrating Kazakhstan into international *DMD* therapeutic networks.

**Figure 7:**
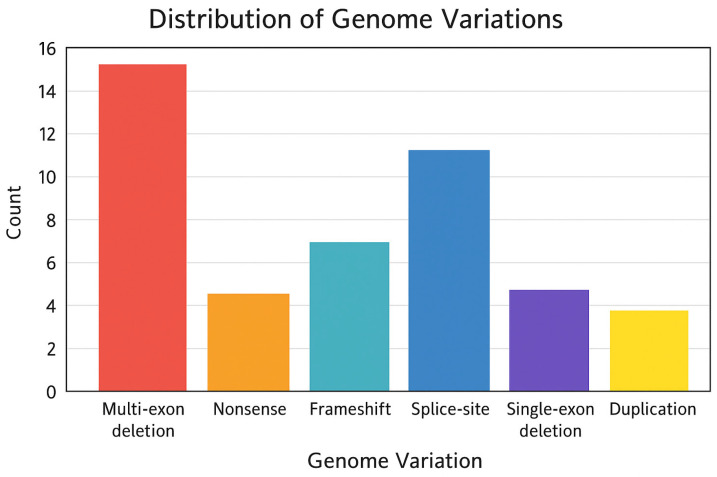
Distribution of identified genome variations in the *DMD* gene. The bar chart summarises the frequency of major variant categories detected in the cohort, including multi-exon deletions, nonsense variants, frameshift variants, splice-site alterations, single-exon deletions, and duplications. Multi-exon deletions constitute the most significant proportion of all observed genomic alterations, followed by splice-site and frameshift variants. These data illustrate the heterogeneous but non-random distribution of pathogenic mechanisms underlying dystrophin deficiency. Blue bars indicate frameshift variants resulting from small nucleotide insertions or duplications that generate premature termination codons.

The distribution of genome variation types shows a clear predominance of multi-exon deletions, which is consistent with the known structural instability of the central region of the *DMD* gene. Splice-site and frameshift variants also represent substantial contributors, reflecting the gene’s susceptibility to both large-scale rearrangements and small-scale sequence disruptions. Nonsense variants, single-exon deletions, and duplications occur less frequently but still contribute to the diverse mutational spectrum typical of dystrophinopathies. The distribution observed in this cohort aligns with global patterns and supports the subsequent genotype–phenotype modelling performed in the study (See Figure 7). Unlike earlier figures that emphasise exon distribution or clinical impact, Figure 7 illustrates the relative contribution of distinct genomic mechanisms—such as multi-exon deletions, frameshift insertions, splice-site alterations, and duplications—to the overall mutation spectrum in the cohort.

**Figure 8:**
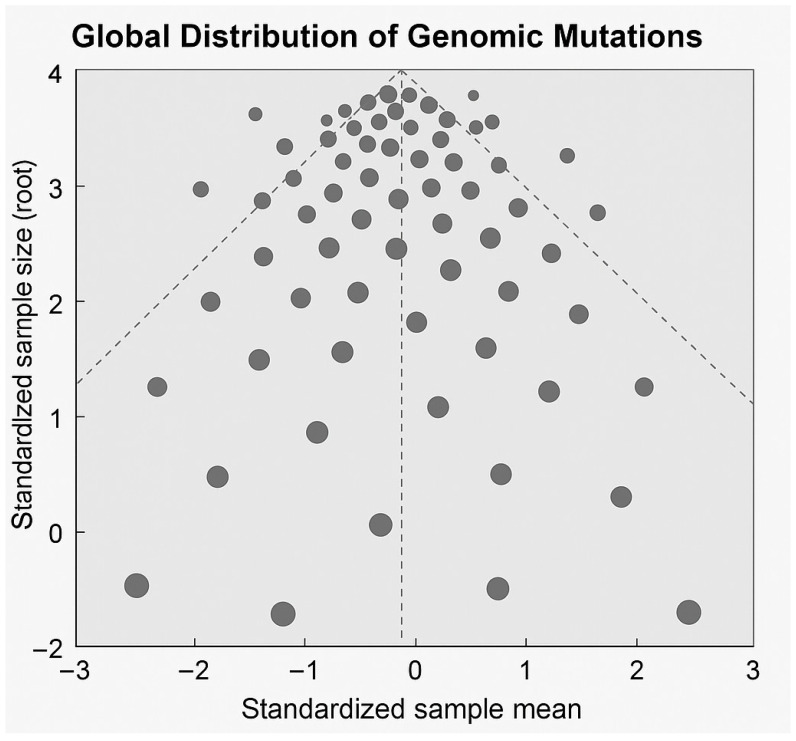
Funnel plot illustrating the global distribution of *DMD* gene mutations and their association with clinical phenotype. The funnel plot displays the dispersion of identified genomic variants across the cohort, explaining their relative distribution around a central mean effect. Each point represents an individual mutation event, with vertical alignment indicating deviation from the mean and horizontal spread reflecting variability in mutational characteristics. The symmetric funnel boundaries visualise expected variation and highlight regions of clustering corresponding to recurrent or hotspot-associated variants. This representation provides an overview of the non-uniform genomic architecture underlying dystrophinopathies.

The funnel plot (Figure 8) offers a global perspective on the distribution of *DMD* gene mutations within the dataset. Most variants fall within the funnel’s central region, indicating consistency with expected mutational variance. At the same time, the concentration of points near the upper centre suggests recurrent events or hotspot-associated clustering—patterns characteristic of the deletion-prone central portion of the *DMD* gene. Wider dispersion toward the lower region reflects less frequent, heterogeneous mutation types such as rare splice-site disruptions or small duplications. This visualisation supports earlier findings from exon-mapping and mutation-spectrum analyses, confirming that the cohort’s mutation landscape follows known global patterns while preserving distinctive distributional features. Each point represents an individual pathogenic or likely pathogenic variant mapped across the *DMD* gene. Variants clustering within the central hotspot region (exons 44–55) and those showing greater deviation from the mean are predominantly associated with severe phenotypes, including earlier loss of ambulation. In contrast, variants distributed toward the funnel periphery are more frequently linked to milder clinical courses, such as preserved ambulation or delayed disease progression. This figure provides an integrated visual representation of the relationship between mutation distribution and functional outcome. In addition, points outside the central funnel essentially correspond to mutations associated with earlier loss of ambulation. In contrast, centrally clustered variants are more often observed in patients with milder or intermediate phenotypes.

The “standardised sample mean” was computed as a z-score:

$$z_{i}=\frac{x_{i}-\bar{x}}{s}$$

For each exon, the standardised score is calculated by comparing its mutation count to the average mutation count across all exons, accounting for typical variation observed in the data. This score represents how many standard deviations the exon’s mutation burden is above or below the overall mean. A score of zero indicates that the exon has a mutation burden exactly equal to the average. A positive score signifies an above-average mutation burden, reflecting enrichment or hotspot activity. Conversely, a negative score indicates a below-average mutation burden for that exon.

### 3.4. Comparison with Phenotype

Across the cohort, a consistent genotype–phenotype gradient emerged, wherein multi-exon deletions—particularly those involving the canonical hotspot between exons 44–55—were the most frequent pathogenic mechanism and corresponded to the earliest loss of ambulation, the steepest decline on Kaplan–Meier survival curves, and some of the highest serum CPK values, often exceeding 10,000 U/L, confirming their strong association with severe dystrophinopathy; nonsense and frameshift variants also produced substantial protein disruption and clustered in the moderate-to-severe phenotype range, although their clinical trajectories were more heterogeneous, with early-exon truncating variants causing rapid functional decline while later-exon events showed milder progression, reflecting known exceptions to the reading-frame rule; splice-site mutations demonstrated a consistently milder clinical course, with all affected patients remaining ambulatory at last follow-up and exhibiting only moderately elevated CPK, suggesting partial preservation of dystrophin expression; duplications represented the mildest mutation class in the entire dataset, as all individuals harbouring them were ambulatory with comparatively lower CPK levels and maintained the highest survival probabilities; collectively, CPK levels correlated positively with the number of exons affected, yet extreme elevations were not absolute predictors of early functional loss, underscoring biochemical–clinical dissociation in some patients; pathogenicity classifications aligned closely with observed outcomes, with multi-exon deletions overwhelmingly designated as pathogenic, while sequence-level alterations—including nonsense, frameshift, and splice-site variants—more frequently fell into the likely pathogenic category, matching their variable clinical severity; integrated analysis across mutation class, pathogenicity, exon location, and biochemical markers confirmed a robust and biologically coherent pattern in which the extent and nature of genomic disruption strongly shaped dystrophin deficiency severity, producing a phenotype hierarchy that ranged from severe truncating and hotspot deletions to intermediate nonsense and frameshift events and finally to the mild profiles seen in splice-site variants and duplications. The recurrence of deletions spanning exons 45–50 and 46–50 across unrelated patients is consistent with the established deletion-prone architecture of the *DMD* hotspot region (exons 44–55). While this pattern may reflect regional clustering in our cohort, the present dataset does not allow inference of a founder effect, which would require haplotype analysis and/or familial segregation data. Future studies should include haplotype-based analyses and, where possible, family-based segregation analyses to determine whether any recurrent deletions reflect shared ancestry or recurrent rearrangements driven by local genomic architecture.

## 4. Discussion

This study provides the first comprehensive statistical-genetics assessment of *DMD* gene mutations in a Kazakh cohort, integrating MLPA- and NGS-confirmed variants with ambulation outcomes, biochemical profiles, and survival modelling. The results demonstrate that the mutational architecture of dystrophinopathy in Kazakhstan broadly reflects global patterns, while simultaneously revealing population-specific features that may inform future diagnostic and therapeutic strategies.

Consistent with large international datasets, multi-exon deletions constituted the predominant mutation class. They concentrated within the canonical hotspot spanning exons 44–55, which has been widely reported as the most deletion-prone region of the *DMD* gene [5,6]. The recurrence of deletions affecting exons 46–50 and 45–50 across unrelated families supports the established structural vulnerability of this genomic segment. Still, it may also suggest regional mutational clustering or shared ancestral haplotypes within the Kazakh population. This trend aligns with previous studies documenting ethnic and geographical variation in *DMD* mutation spectra, particularly in genetically homogeneous or partially isolated populations. Notably, several patients in our cohort carried substantial deletions such as exons 1–62 or 22–44—genotypes typically associated with severe Duchenne phenotypes in global cohorts—but some of these individuals remained ambulatory, indicating phenotypic deviation from classical expectations. Such atypically mild trajectories raise the possibility of local protective modifiers, whether genetic, epigenetic, or environmental, and highlight an essential direction for future functional genomics work in Central Asia.

The phenotype hierarchy observed in this study—where hotspot-associated deletions and early-truncating variants produced the most severe outcomes, while splice-site variants and duplications resulted in milder trajectories—is consistent with the established “reading-frame rule” [7] and corroborates known exceptions [8,9,10]. Early-exon nonsense and frameshift variants, such as p. Lys1049* and p. Ser861Ilefs*7, were associated with rapid loss of ambulation. In contrast, later-exon truncations demonstrated more variable courses, reinforcing prior reports that the location of the truncation and the potential for exon skipping or alternative splicing strongly influence clinical severity. The relatively benign phenotype observed in splice-site and duplication carriers is consistent with reports indicating partial dystrophin preservation or in-frame rescue in these variant types. Together, these findings validate the biological coherence of the cohort and demonstrate that clinically meaningful genotype–phenotype relationships can be robustly extracted even from moderate-sized cohorts.

Biochemical data in the present study further support this interpretation. Serum CPK values showed broad variability but correlated positively with the number of exons affected. Although several individuals with markedly elevated CPK (>10,000 U/L) remained ambulatory, the overall pattern reflects the cumulative myofiber damage associated with extensive deletions and early-truncating mutations. The disconnect between biochemical and functional severity in some patients underscores the limitations of CPK as a prognostic marker. It reinforces the value of integrating genomic and statistical survival modelling when predicting clinical trajectories.

Statistical modelling revealed that hotspot involvement and out-of-frame consequences were strong independent predictors of early ambulation loss, consistent with prior research demonstrating the prognostic value of these features in dystrophinopathy [7,14]. Kaplan–Meier survival curves effectively visualised this hierarchy, with deletions and truncating variants showing the earliest decline, and splice-site or duplication carriers maintaining high long-term probabilities of ambulation. These findings demonstrate the utility of incorporating survival analysis and regression modelling into routine dystrophinopathy genetic reporting, particularly in settings where longitudinal clinical data are limited or heterogeneous.

A broader implication of this work lies in its relevance to emerging mutation-targeted therapies. A substantial proportion of the cohort carried variants potentially amenable to exon-skipping approaches targeting exons 45, 51, or 53, or nonsense readthrough therapy. These observations highlight a significant untapped opportunity for implementing precision-medicine strategies in Kazakhstan once access to molecular therapies expands. Furthermore, the presence of recurring variant types across unrelated families suggests that region-specific diagnostic algorithms—for example, prioritising MLPA panels optimised for the 44–55 hotspot—could improve diagnostic efficiency in this population.

Future research directions include (1) expanding cohort size through multi-centre collaboration; (2) performing haplotype analyses to evaluate potential founder effects; (3) investigating putative Kazakh-specific modifier alleles using whole-genome or transcriptome sequencing; and (4) establishing a national dystrophinopathy registry to harmonise genetic, clinical, and therapeutic data. Integration of Kazakhstan into international rare-disease networks and clinical trials will also be essential to harness the therapeutic potential of exon-skipping and gene-based treatments.

Taken together, this study demonstrates that while the Kazakh dystrophinopathy spectrum adheres to global genomic principles, it also contains distinctive features—such as recurrent multi-exon deletions, unusually mild presentations of large-scale deletions, and unique splice-site events—that warrant deeper investigation. By placing these findings within the broader context of neuromuscular genomics, this work lays the foundation for future precision-medicine initiatives in Central Asia. It strengthens the global understanding of *DMD* gene variation.

The mutation spectrum in this cohort has direct therapeutic relevance. Multiple patients carried variants amenable to exon skipping (exons 45, 51, and 53), while those with nonsense mutations represent candidates for stop-codon readthrough therapy. Recurrent multi-exon deletions and hotspot localisation further correspond to established therapeutic windows for PMO/ASO-based interventions and emerging CRISPR exon-editing platforms. These findings demonstrate that a substantial fraction of Kazakh patients is theoretically eligible for RNA-targeted or mutation-specific therapies, underscoring the importance of integrating advanced molecular diagnostics, national *DMD* registries, and precision-medicine infrastructure into Kazakhstan’s neuromuscular care strategy

- Exon skipping (45/51/53): 30 patients”
- “Stop-codon readthrough: 15 patients”
- “Gene replacement (micro-dystrophin): 25 patients”
- “Gene editing (exon rescue): 25 patients”

A critical translational dimension of the present dataset lies in its alignment with established therapeutic windows for mutation-specific interventions. Based on the observed genotypes, 30 patients (the majority of the cohort) carried deletions amenable to exon skipping targeting exons 45, 51, or 53. In comparison, 15 patients harboured nonsense mutations suitable for stop-codon readthrough therapy. An additional 25 patients were candidates for micro-dystrophin gene-replacement approaches, and 25 patients carried variant types positioned for future CRISPR-mediated exon-rescue strategies. These proportions closely reflect the underlying mutation spectrum—dominated by hotspot-associated multi-exon deletions and early-truncating variants—and reinforce the idea that therapeutic eligibility is directly shaped by genomic architecture in this population.

The therapeutic windows defined by the FDA-approved exon-skipping agents—eteplirsen (exon 51), golodirsen/viltolarsen (exon 53), and casimersen (exon 45)—correspond precisely to the most frequently disrupted regions in our cohort, particularly deletions spanning exons 45–50 or 46–50. This convergence between mutation hotspots and therapeutic hotspots is a key translational finding: it suggests that a significant fraction of Kazakh patients would qualify for exon-skipping approaches immediately if these treatments were accessible. Likewise, the confirmed nonsense variants (e.g., p.Lys1049*), although fewer in number, represent a well-defined group for ataluren-mediated readthrough therapy, which already has regulatory use in other regions.

The landscape of RNA-targeting therapeutics continues to expand, with phosphorodiamidate morpholino oligomers (PMOs), antisense oligonucleotides (ASOs), and next-generation peptide-conjugated PMOs (PPMOs) progressing through regulatory pathways. Recent approvals and late-phase trials underscore the growing maturity of RNA-based approaches for *DMD*, particularly for exon skipping. Emerging modalities—including CRISPR/Cas9 exon excision, splice-modulating ASOs, and transcript-stabilising strategies—are increasingly designed with fine-grained mutation specificity, reinforcing the clinical relevance of detailed exon-level characterisation. The mutation profile identified in this cohort, dominated by deletions in the central hotspot and enriched for frame-disrupting events, aligns well with the mutational targets of these evolving RNA therapeutics.

These therapeutic eligibility patterns have substantial implications for Kazakhstan, where advanced *DMD* treatments are not yet widely available. The data presented here demonstrate that a meaningful proportion of patients are already positioned for mutation-specific therapy and could therefore benefit immediately from exon-skipping or readthrough-based interventions upon regulatory or economic approval. This information is vital for:

National health planning—forecasting the expected number of candidates for exon-skipping biologics or gene therapy.

Clinical-trial readiness—identifying which patients could be rapidly enrolled in multinational *DMD* trials.

Diagnostic pathway optimisation—prioritising MLPA and NGS pipelines that emphasise the 44–55 hotspot and the early-truncation zones most relevant to exon-skipping therapies.

Precision-medicine infrastructure building—laying the groundwork for implementation of future RNA-based and gene-editing treatments

Given that the Kazakh mutation spectrum mirrors global patterns while retaining regional features—such as recurrent deletions in unrelated families and unusually mild presentations of large-scale deletions—this population represents both an essential inclusion in international *DMD* therapeutic networks and a potential source of unique biological insight, including modifier alleles or splicing patterns that may influence drug response.

Taken together, integrating mutation-specific therapy eligibility into the genotype framework presented in this study enhances the translational value of the findings. The predominance of exon-skipping–amenable deletions, coupled with the presence of nonsense variants and early-truncating mutations, positions Kazakhstan for future adoption of RNA-targeting therapies and gene-based interventions. These insights underscore the necessity of establishing national dystrophinopathy registries, harmonised diagnostic guidelines, and collaborative links with global therapeutic development programmes to ensure equitable access to emerging *DMD* treatments in Central Asia.

Beyond mutation-targeted therapies, respiratory viral infections represent a crucial public-health risk in dystrophinopathies because of progressive respiratory insufficiency and cardiomyopathy. In this context, antiviral drug-repurposing studies (e.g., Khaidarov et al., 2025, demonstrating in vitro anti–SARS-CoV-2 activity of tenofovir alafenamide) are cited as general examples of evolving antiviral strategies relevant to vulnerable cardiopulmonary populations; however, clinical efficacy and safety in *DMD* have not been established, and no *DMD*-specific therapeutic conclusions are drawn from this reference [21]. The applicability of such findings to *DMD* is substantial: patients with progressive muscle degeneration and respiratory insufficiency face disproportionate risks from respiratory viral illnesses, and persistent post-viral inflammatory states may exacerbate muscle weakness, cardiac strain, or rehabilitation instability. Integrating emerging antiviral and immunomodulatory strategies—such as those identified by Khaidarov et al.—into *DMD* clinical frameworks may therefore mitigate infection-driven deterioration and reduce hospitalisation rates, particularly in regions such as Kazakhstan, where access to advanced neuromuscular therapies remains limited. These insights highlight the importance of expanding both genetic precision medicine and public-health preparedness, ensuring that *DMD* patients benefit not only from mutation-specific interventions but also from evolving antiviral therapeutics that can meaningfully improve long-term outcomes.

Study limitations and selection bias. This study is retrospective and based on clinically referred patients with genetically confirmed dystrophinopathy. As a result, the cohort is subject to ascertainment (selection) bias toward more severe or clinically apparent cases who were more likely to undergo genetic testing and be captured in hospital records. Consequently, milder phenotypes, including less symptomatic Becker muscular dystrophy presentations and undiagnosed individuals in the community, may be underrepresented. The mutation-class frequencies and genotype–phenotype patterns reported here therefore reflect a diagnostically ascertained cohort and should not be interpreted as estimates of population-level prevalence or the full clinical spectrum of dystrophinopathy in Kazakhstan.

Serum creatine phosphokinase (CPK) is a sensitive biomarker of muscle membrane instability in dystrophinopathy, but it is an imperfect surrogate for long-term functional prognosis [22,23,24]. Significantly, CPK activity varies with age and disease stage: levels typically peak early and tend to decline with progression, in part because ongoing myofibre loss and replacement by fibrofatty tissue reduce the available muscle mass that can release the enzyme into the circulation [22,23,24]. Consequently, very high CPK may be observed in ambulant patients with substantial viable muscle undergoing active damage, whereas lower (or declining) CPK can occur later despite worsening weakness—providing a mechanistic explanation for the biochemical–clinical dissociation observed in some individuals [22,23,24].

Beyond stage effects, functional outcomes are shaped by interindividual differences in inflammatory and fibrotic remodelling and by secondary genetic modifiers that influence muscle repair, fibrosis, and immune signalling [22,23,24]. Variants in LTBP4 (TGF-β pathway) have been associated with age at loss of ambulation, supporting a fibrosis-modifying mechanism that can shift functional trajectories independent of primary *DMD* mutation class [25]. Likewise, the SPP1 (osteopontin) promoter polymorphism has been reported as a determinant of severity/earlier loss of ambulation in some cohorts, consistent with osteopontin’s role in immune activation and tissue remodelling. However, modifier effects have not been uniformly replicated across all populations—highlighting context dependence and the need for population-specific validation [26,27]. Additional modifier signals have been reported in immune-related pathways (e.g., CD40/NF-κB) and in muscle structural/functional genes (e.g., ACTN3), both of which can alter strength and functional performance measures [28,29,30].

Public-health considerations in rare dystrophinopathies. Beyond mutation-targeted approaches, infectious respiratory illnesses are a clinically crucial public health risk in dystrophinopathies because progressive respiratory muscle weakness and cardiomyopathy reduce cardiopulmonary reserve and increase vulnerability to complications during viral infections [31,32,33]. This is particularly relevant in real-world care, where exposure to seasonal respiratory viruses (and, recently, SARS-CoV-2) may precipitate decompensation, accelerate functional decline through inactivity and systemic inflammation, and increase the burden of hospitalisation in a small but high-risk population [22,23]. In this context, Reference [21] (Khaidarov et al., 2025) is cited not as evidence for antiviral treatment in *DMD*, but as an example of ongoing antiviral drug-repurposing work (including testing against locally circulating strains) that may inform broader preparedness for vulnerable cardiopulmonary patient groups [21]. We emphasise that the antiviral findings in Ref. [21] are in vitro and do not establish clinical efficacy or safety in dystrophinopathy; therefore, any relevance to *DMD* should be interpreted as hypothesis-generating and supportive of the need for dedicated clinical studies and integrated public-health planning (vaccination, early respiratory assessment, and guideline-based respiratory management) for rare neuromuscular diseases [21,31,32,33].

## 5. Conclusions

This study concludes the first integrated genomic and statistical-genetics analysis of *DMD* mutations in a Kazakh cohort, revealing a mutational landscape that aligns with global patterns of dystrophinopathy while displaying distinct regional features. Multi-exon deletions within the 44–55 hotspot and early truncating variants were confirmed as the primary drivers of severe phenotypes, whereas splice-site alterations and duplications exhibited consistently milder clinical courses. The combination of MLPA/NGS diagnostics with survival modelling demonstrated strong genotype–phenotype structure, enabling more precise prediction of ambulation outcomes and reinforcing the clinical utility of mutation-level stratification. Notably, recurrent large-scale deletions and unexpectedly mild presentations of typically severe genotypes suggest the presence of potential Kazakh-specific modifier factors, warranting further genomic investigation. These findings strengthen the foundation for implementing mutation-targeted therapies and highlight the importance of establishing regional *DMD* registries and diagnostic pathways to support precision-medicine strategies across Central Asia.

## Figures and Tables

**Table 1:** Cohort Characteristics of Patients with Genetically Confirmed Dystrophinopathy.

Characteristic	Value
Total number of genetically confirmed patients (N)	30
Sex	100% male
Variant type	Deletions 66.7%; SNV 30.0%; Duplications 3.3%
Hotspot region	Exons 45–55
Mutation-negative cases (MLPA)	2 (not included in primary cohort)
Study design	Retrospective cohort

## Data Availability

The data presented in this study are available upon reasonable request from the corresponding author. The dataset contains sensitive clinical and genetic information. It cannot be made publicly available due to ethical and privacy restrictions imposed by the Local Bioethics Committee of Kazakh National Medical University (Protocol No. 01-040325) and the institutional approval from the Republican Children’s Clinical Hospital “Aksai”. Only fully anonymised and depersonalised data can be shared, and only in compliance with Kazakh national regulations governing the protection of patient genetic information. No new publicly archived datasets were generated during this study.

## Abbreviations

The following abbreviations are used in this manuscript:

ACMG	American College of Medical Genetics and Genomics
BMD	Becker muscular dystrophy
CAP	College of American Pathologists
CK/CPK	Creatine kinase/Creatine phosphokinase
CLIA	Clinical Laboratory Improvement Amendments
cDNA	Complementary DNA
DMD	Duchenne muscular dystrophy/*DMD* gene
gDNA	Genomic DNA
HGMD	Human Gene Mutation Database
IQR	Interquartile range
IRB	Institutional Review Board
KM	Kaplan–Meier
LOA	Loss of ambulation
MLPA	Multiplex ligation-dependent probe amplification
NGS	Next-generation sequencing
PCR	Polymerase chain reaction
RNA	Ribonucleic acid
SD	Standard deviation
SPSS	Statistical Package for the Social Sciences

## References

[1] Koenig M., Hoffman E.P., Bertelson C.J., Monaco A.P., Feener C., Kunkel L.M. (1987). Complete cloning of the Duchenne muscular dystrophy (DMD) cDNA and preliminary genomic organization of the DMD gene. Cell.

[2] Hoffman E.P., Brown R.H., Kunkel L.M. (1987). Dystrophin: The protein product of the Duchenne muscular dystrophy locus. Cell.

[3] Aartsma-Rus A., Ginjaar I.B., Bushby K. (2016). The importance of genetic diagnosis for Duchenne muscular dystrophy. J. Med. Genet..

[4] Emery A.E.H. (2002). The muscular dystrophies. Lancet.

[5] Bladen C.L., Salgado D., Monges S., Foncuberta M.E., Kekou K., Kosma K., Dawkins H., Lamont L., Roy A.J., Chamova T. (2015). The TREAT-NMD DMD Global Database: Analysis of more than 7,000 Duchenne muscular dystrophy mutations. Hum. Mutat..

[6] Takeshima Y., Yagi M., Okizuka Y., Awano H., Zhang Z., Yamauchi Y., Nishio H., Matsuo M. (2010). Mutation spectrum of the dystrophin gene in Japanese patients with Duchenne/Becker muscular dystrophy. Hum. Genet..

[7] Monaco A.P., Bertelson C.J., Liechti-Gallati S., Moser H., Kunkel L.M. (1988). An explanation for the phenotypic differences between patients bearing partial deletions of the DMD locus. Genomics.

[8] Zimowski J., Cegielska J., Rzońca T., Ryniewicz B., Kaczorowska M., Zaremba J., Kostera-Pruszczyk A., Jędrzejowska M., Fidziańska A., Hausmanowa-Petrusewicz I. (2020). Phenotype variability in dystrophinopathies: Exceptions to the reading-frame rule. Neurol. Neurochir. Pol..

[9] Flanigan K.M. (2014). Duchenne and Becker muscular dystrophies. Neurol. Clin..

[10] Bello L., Pegoraro E. (2018). The “reading-frame rule” in Duchenne muscular dystrophy: Still valid?. Neuromuscul. Disord..

[11] White S., Aartsma-Rus A., Flanigan K., Weiss R., Kneppers A., Lalic T., Janson A., Ginjaar H., Breuning M., Dunnen J.D. (2006). Duplications in the DMD gene. Hum. Mutat..

[12] Ankala A., Kohn J.N., Hegde S. (2015). Dystrophinopathies: Clinical and genetic testing strategies. Neurol. Genet..

[13] Lim K.R.Q., Sheri R., Yokota T. (2020). Exon skipping applications in Duchenne muscular dystrophy. Int. J. Mol. Sci..

[14] Aartsma-Rus A., Straub V., Hemmings R., Haas M., Schlosser-Weber G., Stoyanova-Beninska V., Mercuri E., Muntoni F., Sepodes B., Vroom E. (2017). Development of exon skipping therapies for Duchenne muscular dystrophy: A critical review. Lancet Neurol..

[15] Mendell J.R., Sahenk Z., Lehman K., Nease C., Lowes L.P., Miller N.F., Iammarino M.A., Alfano L.N., Nicholl A., Al-Zaidy S. (2013). Assessment of the safety and efficacy of ataluren for nonsense mutation DMD. Lancet.

[16] Bushby K., Finkel R., Birnkrant D.J., Case L.E., Clemens P.R., Cripe L., Kaul A., Kinnett K., McDonald C., Pandya S. (2010). Diagnosis and management of Duchenne muscular dystrophy, part 1. Lancet Neurol..

[17] Schouten J.P., McElgunn C.J., Waaijer R., Zwijnenburg D., Diepvens F., Pals G. (2002). MLPA: A novel multiplex PCR method for detection of deletions/duplications. Nucleic Acids Res..

[18] Richards S., Aziz N., Bale S., Bick D., Das S., Gastier-Foster J., Grody W.W., Hegde M., Lyon E., Spector E. (2015). Standards and guidelines for the interpretation of sequence variants (ACMG-AMP). Genet. Med..

[19] Balding D.J. (2006). A tutorial on statistical genetics. Nat. Rev. Genet..

[20] Goeman J.J., Solari A. (2014). Multiple testing and survival analysis. Stat. Med..

[21] Khaidarov S., Hejran A.B., Moldakaryzova A., Izmailova S., Nurgaliyeva B., Beisenova A., Mustafaeva A., Nurzhanova K., Belova Y., Satbayeva E. (2025). An Anti-HIV Drug Is Highly Effective Against SARS-CoV-2 In Vitro and Has Potential Benefit for Long COVID Treatment. Viruses.

[22] Kim E.Y., Lee J.W., Suh M.R., Choi W.A., Kang S.W., Oh H.J. (2017). Correlation of Serum Creatine Kinase Level With Pulmonary Function in Duchenne Muscular Dystrophy. Ann. Rehabil. Med..

[23] Sun S.-C., Peng Y.-S., He J.-B. (2008). Changes of serum creatine kinase levels in children with Duchenne muscular dystrophy. Zhongguo Dang Dai Er Ke Za Zhi.

[24] Zatz M., Rapaport D., Vainzof M., Passos-Bueno M.R., Bortolini E.R., Pavanello R.d.C.M., Peres C.A. (1991). Serum creatine-kinase (CK) and pyruvate-kinase (PK) activities in Duchenne (DMD) as compared with Becker (BMD) muscular dystrophy. J. Neurol. Sci..

[25] Flanigan K.M., Ceco E., Lamar K., Kaminoh Y., Dunn D.M., Mendell J.R., King W.M., Pestronk A., Florence J.M., Mathews K.D. (2013). LTBP4 genotype predicts age of ambulatory loss in Duchenne muscular dystrophy. Ann. Neurol..

[26] Pegoraro E., Hoffman E., Piva L., Gavassini B., Cagnin S., Ermani M., Bello L., Soraru G., Pacchioni B., Bonifati M.D. (2011). SPP1 genotype is a determinant of disease severity in Duchenne muscular dystrophy. Neurology.

[27] Kosac A., Pesovic J., Radenkovic L., Brkusanin M., Radovanovic N., Djurisic M., Radivojevic D., Mladenovic J., Ostojic S., Kovacevic G. (2022). LTBP4, SPP1, and CD40 Variants: Genetic Modifiers of Duchenne Muscular Dystrophy Analyzed in Serbian Patients. Genes.

[28] Bello L., Flanigan K.M., Weiss R.B., Dunn D.M., Swoboda K.J., Gappmaier E., Howard M.T., Sampson J.B., Bromberg M.B., Butterfield R. (2016). Association Study of Exon Variants in the NF-κB and TGFβ Pathways Identifies CD40 as a Modifier of Duchenne Muscular Dystrophy. Am. J. Hum. Genet..

[29] Hogarth M.W., Houweling P.J., Thomas K.C., Gordish-Dressman H., Bello L., Pegoraro E., Hoffman E.P., Head S.I., North K.N. (2017). Evidence for ACTN3 as a genetic modifier of Duchenne muscular dystrophy. Nat. Commun..

[30] Barnard A.M., Hammers D.W., Triplett W.T., Kim S., Forbes S.C., Willcocks R.J., Daniels M.J., Senesac C.R., Lott D.J., Arpan I. (2022). Evaluating Genetic Modifiers of Duchenne Muscular Dystrophy Disease Progression Using Modeling and MRI. Neurology.

[31] Childs A.-M., Turner C., Astin R., Bianchi S., Bourke J., Cunningham V., Edel L., Edwards C., Farrant P., Heraghty J. (2024). Development of respiratory care guidelines for Duchenne muscular dystrophy. Thorax.

[32] Schultz T.I., Raucci F.J., Salloum F.N. (2022). Cardiovascular Disease in Duchenne Muscular Dystrophy. J. Clin. Med..

[33] Sales T.L.S., Pereira D.N., Gomes V.M.R., de Aguiar G.G., Marcolino M.S. (2025). Efficacy of tenofovir on clinical outcomes of COVID-19: A review of clinical trials. BMC Infect. Dis..

